# Concomitant Multidrug-resistant Pulmonary Tuberculosis and Susceptible Tuberculous Meningitis

**DOI:** 10.3201/eid2003.131205

**Published:** 2014-03

**Authors:** Christine Bernard, Florence Brossier, Mathilde Fréchet-Jachym, Philippe C. Morand, Sophie Coignard, Elisabeth Aslangul, Alexandra Aubry, Vincent Jarlier, Wladimir Sougakoff, Nicolas Veziris

**Affiliations:** Université Pierre et Marie Curie, Paris, France (C. Bernard, F. Brossier, A. Aubry, V. Jarlier, W. Sougakoff, N. Veziris);; Hôpital Pitié-Salpêtrière, Paris (C. Bernard, F. Brossier, A. Aubry, V. Jarlier, W. Sougakoff, N. Veziris);; Centre Médical de Bligny, Briis sous Forges, France (M. Fréchet-Jachym);; Groupe Hospitalier Cochin, Paris (P. C. Morand, S. Coignard); Université Paris Descartes, Paris (P. C. Morand);; Hôpital Hôtel-Dieu, Paris (E. Aslangul)

**Keywords:** Mycobacterium tuberculosis, tuberculosis, drug resistance, heterogenous infection, compartmentalization, tuberculosis and other mycobacteria, France, Romania

**To the Editor:** In 2012, a 34-year-old HIV-seronegative man was hospitalized after several months of cough, fever, night sweats, 10-kg weight loss, and, in the past month, severe headache. The patient was born in Romania and had lived in France for 2 years. He had a history of pulmonary tuberculosis (TB) for which treatment was started in Romania in 2006 and 2008, but he did not complete treatment. The treatment he received in Romania was unknown.

At hospital admission, the patient had a fever of 39°C, stiff neck, and swollen cervical and axillary lymph nodes. A chest radiograph showed multiple cavities and nodular opacities in both superior lobes. Sputum auramine staining indicated that acid-fast bacilli was positive, which supported the diagnosis of pulmonary TB. Examination of cerebrospinal fluid (CSF) revealed hypoglycorrachia (0.95 mmol/L, concentration ratio CSF/blood: 0.2 [reference range 0.5–0.75]), hyperproteinorrachia (1.3 g/L [reference range 0.2–0.4 g/L]), erythrocyte count 2,000 μL (reference value <10 μL), and leukocyte count 150 μL (reference value <10 μL). Auramine staining showed no acid-fast bacilli in CSF. Standard antituberculous therapy with rifampin (RIF), isoniazid (INH), pyrazinamide, and ethambutol was started.

Genomic amplification–based assay (Xpert MTB/RIF; Cepheid, Maurens-Scopont, France), performed on sputum, confirmed the presence of the *Mycobacterium tuberculosis* genome and detected resistance to RIF (Table). The line probe assay Genotype MTBDR*plus* (Hain Lifescience, Bandol, France) performed on sputum showed a positive signal for all wild-type sequences and for *rpoB* (S531L associated with RIF resistance) and *katG* (S315T associated with INH resistance) mutations, suggesting the presence of mixed susceptible and resistant *M. tuberculosis*. Second-line treatment was started: moxifloxacin, amikacin, ethionamide, para-aminosalicylic acid, cycloserine and linezolid. The presence of mixed *M. tuberculosis* organisms in lungs was confirmed with culture methods and by phenotypic drug susceptibility testing (DST) that showed 1% resistant mutant to RIF and INH (proportion method) (*1*). The isolate was considered resistant to RIF and INH and was thus categorized as multidrug resistant (MDR). DST also showed that the sputum isolate had an elevated proportion of ofloxacin-resistant mutants (2 mg/L, 0.02% resistant mutants). Although no mutation in *gyrA* or *gyrB* was detected on colonies grown in the absence of fluoroquinolone, a *gyrB* N538D mutation was identified on colonies grown on ofloxacin medium. On the basis of the DST results, the treatment was changed to ethambutol, pyrazinamide, amikacin, moxifloxacin, para-aminosalicylic acid, and linezolid. CSF culture was eventually positive for *M. tuberculosis* on Lowenstein-Jensen medium after 30 days. DST performed on the CSF isolate showed a drug-sensitive phenotype; thus, RIF was reintroduced in addition to the other antimicrobial drugs. After 3 months, although the patient had improved, he left the hospital against medical advice without providing follow-up contact information.

**Table:** Localization, drug-susceptibility results, and MIRU-VNTR genotypes of *Mycobacterium tuberculosis* strains recovered from a man with both pulmonary tuberculosis and tuberculous meningitis, France, 2012*

Tissue	MIRU-VNTR genotype 1 222213222234225153333622	MIRU-VNTR genotype 2 224243122234225143335622
Lung	Isoniazid and rifampin resistant, fluoroquinolone susceptible, *rpoB* S531L, *katG* S315T, no mutations in *gyrA* and *gyrB*	Pan-susceptible except to fluoroquinolones, no mutations in *rpoB*, *katG,* and *gyrA*; *gyrB* N538D
Cerebrospinal fluid		Pan-susceptible, no mutations in *rpoB*, *katG*, *gyrA*, and *gyrB*
*MIRU-VNTR, mycobacterial interspersed repetitive unit–variable number tandem repeat.		

Mycobacterial interspersed repetitive unit–variable number tandem repeat (MIRU-VNTR) analysis was conducted on culture of the sputum and CSF sample and on colonies grown on INH, RIF, and ofloxacin–containing medium, as described (*2*) (Table). This analysis showed different genotypes for the lung MDR and the CSF-susceptible isolates. None of the MIRU-VNTR patterns were linked to known lineages. The mutation rate of MIRU-VNTR has been evaluated at 2.7 × 10–3/loci/year (*3*). Rather than natural evolution of 1 strain, the 5-locus difference observed for the MIRU-VNTR genotypes is probably attributable to infection with 2 *M. tuberculosis* strains. Surprisingly, MIRU-VNTR patterns were identical for the *gyrB* mutated lung isolate and to the antimicrobial drug–susceptible isolate obtained from CSF. Also, the *gyrB* mutated lung isolate was susceptible to other antimicrobial drugs, whereas the lung MDR isolate did not harbor any *gyrB* mutation. Taken together, both genotype and DST heterogeneity were shown (Table). None of the drug-resistant clones were isolated in CSF.

This heterogenous infection raises 2 hypotheses: the patient was infected with both the MDR and the non-MDR strains or with 2 non-MDR strains and acquired additional drug resistance in the lung during treatment. Clonal differences among *M. tuberculosis* culture isolates obtained from pulmonary and CSF samples indicate compartmentalization (*4*). Heterogeneity in *M. tuberculosis* isolates already has been reported either for genotypes or for drug susceptibility (*5*–*7*). However, combination of genotype and resistance heterogeneity is a diagnostic and therapeutic challenge (*8*). In our laboratory, the line probe assay performed directly on sputum detected the 1% INH- and RIF-resistant mycobacteria, although a previous report suggest that this assay could not detect <5% resistant mycobacteria (*9*). The antimicrobial drug–resistant clones were isolated from only lung. The higher bacillary population in lungs than in central nervous system may account for an increased selection of resistant mutants in lungs. This finding raises the question of whether DST obtained from lung isolates should be used for establishing TB treatment at extrapulmonary localizations, especially in patients with meningitis, for whom effective treatment is an emergency. Because first-line treatments are more effective, we wonder whether treatment for MDR TB patients who have secondarily acquired resistance and both lung and central nervous system TB should target MDR and drug-susceptible TB until the CSF strain is proven to also be MDR.
